# Diagnosing a popliteal venous aneurysm in a primary care setting: A case report

**DOI:** 10.1186/1752-1947-2-307

**Published:** 2008-09-22

**Authors:** Emmanouil K Symvoulakis, Spyridon Klinis, Ioannis Peteinarakis, Dimitrios Kounalakis, Nikos Antonakis, Emmanouil Tsafantakis, Christos Lionis

**Affiliations:** 1 Clinic of Social and Family Medicine, Department of Social Medicine, Faculty of Medicine, University of Crete, Heraklion, Crete, Greece; 2 Primary Health Centre of Anogia, Rethymno, Crete, Greece

## Abstract

**Introduction:**

Popliteal venous aneurysms are uncommon but potentially fatal vascular disorders. They can be symptomatic or asymptomatic, mimicking different conditions. Popliteal venous aneurysms are possible sources of embolism.

**Case presentation:**

A 68-year-old woman presented at a rural primary health care unit in Crete, Greece, reporting local symptoms of discomfort in the right popliteal fossa with pain during palpation. Colour Doppler ultrasonography revealed local widening and saccular dilatation in the right distal popliteal vein. The diagnosis of a popliteal venous aneurysm was formulated.

**Conclusion:**

Popliteal venous aneurysms are rare conditions, but are potentially more common than usually thought in daily practice. Physician awareness and access to ultrasound examination may allow for early diagnosis, before the occurrence of any thromboembolic or other major complication.

## Introduction

Popliteal venous aneurysms may cause fatal complications, such as pulmonary embolism and other thromboembolic episodes, [1,2] if they remain undiagnosed or untreated. These lesions may have a more or less symptomatic presentation. A safe management approach lies in surgical repair and therefore the early detection of these conditions is crucial. Few cases of popliteal venous aneurysm are reported worldwide. They are more common in females and occur more frequently in people over 40 years of age [3-6]. We report a case of a 68-year-old woman with popliteal venous aneurysm of the right lower extremity, diagnosed in a primary care setting in rural Crete, as an example of how 'unexplained' local symptoms and adequate work-up can lead to the early diagnosis of a rare condition.

## Case presentation

A 68-year-old woman presented to her general practitioner with a history of local discomfort and swelling in the right popliteal fossa over the previous few months. Symptoms worsened during usual daily activities (walking and climbing stairs). The patient had a history of chronic bilateral venous insufficiency, with its onset after pregnancy. Bilateral saphenectomy (at different time points) was performed after years of suffering. Recurrence occurred in both lower extremities approximately 12 years after surgical management. The patient had a strong family history of varicose veins.

**Figure 1:**
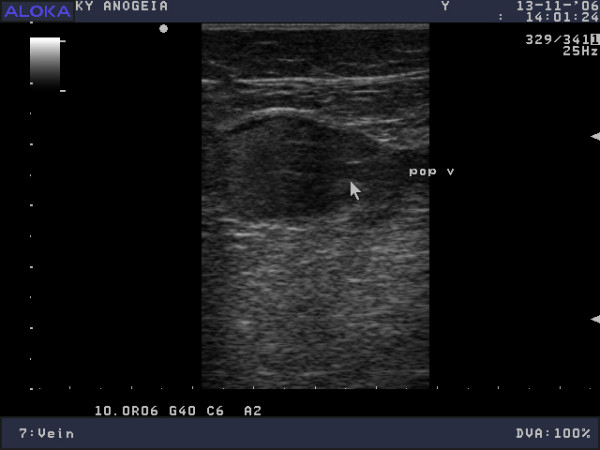
**Distal right popliteal vein, B-mode ultrasonogram, transverse axis**. The vein lumen could be obliterated using a small amount of extrinsic pressure.

**Figure 2:**
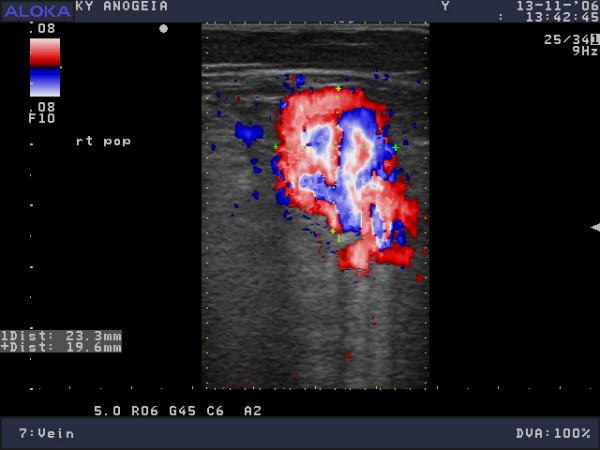
Distal right popliteal vein, colour Doppler ultrasonogram, oblique-transverse axis.

**Figure 3:**
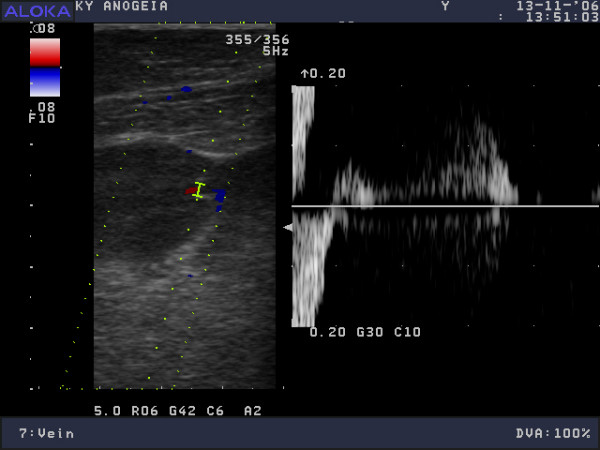
Distal right popliteal vein, colour Doppler ultrasonogram, spectral analysis during the calf muscle squeeze test.

Signs of chronic bilateral venous insufficiency were evident. Physical examination was positive for the presence of a soft mass, painful on deep palpation, in the upper part of the popliteal right fossa with no local signs of inflammation or murmur. Chest and abdomen examination was normal. No evident clinical signs of peripheral arterial angiopathy were detected. Arterial blood pressure, chest X-ray, oxygen saturation and electrocardiogram were normal. Colour Doppler ultrasonography was performed by a qualified radiologist. Real-time B-mode and colour Doppler ultrasonography revealed local widening and saccular dilatation (2.3 × 1.9 × 2.4 cm) in the right distal popliteal vein (Figures 1 and 2). Colour Doppler spectral analysis detected a vein waveform that was altered during a calf-muscle squeeze test (Figure 3). The volume of the lesion slightly increased in size during the Valsalva manoeuvre. Although blood flow within the lesion was slow, there was no evidence of thrombosis in the saccular dilatation (compression test was negative; moreover, the lesion was completely filled with blood during the calf-muscle squeeze test, as depicted using colour Doppler ultrasonography). The right popliteal artery colour Doppler waveform was normal. Medical information was provided to the patient regarding the diagnosis and the option of an urgent referral to specialists was recommended as the next step in care.

## Discussion

Popliteal venous aneurysm can lead to severe complications including deep vein thrombosis, pulmonary emboli and death [7,8]. It was first described as an uncommon cause of pulmonary embolism 30 years ago [2]. Asymptomatic incidental detection, local lower extremity symptoms or embolic pulmonary episodes may represent different aspects of presentation of the same condition [1]. Most cases present as episodes of pulmonary embolism, a potentially life-threatening complication [9]. In the case of our patient, the diagnosis was probably related to an advanced stage of chronic venous insufficiency and strong hereditary conditioning factors.

In the past, the most commonly used diagnostic procedure was phlebography, which has been increasingly replaced by colour Doppler ultrasonography in recent years [10]. There is sufficient evidence to support the suitability of colour Doppler venous scanning in diagnosing popliteal venous aneurysms [10-14]. Ultrasonography of leg vessels is useful as a preliminary detection technique [13], being non-invasive and easily repeatable, with low cost and lacking ionising radiation. Its utility becomes more evident and perhaps unique in a primary care setting. Furthermore, this technique is reliable in detecting the exact aneurysm site, the presence of a thrombus within the aneurysmatic sac, and any coexistent venous anomalies or other disorders such as a Baker's cyst [14], offering useful information for the differential diagnostic procedure. Baker's cyst is a persistent joint fluid effusion (synovial) that forms in the back of the knee or can be caused, more frequently in adults, by posterior herniation of the knee joint capsule. Cysts of the proximal tibiofibular joint are rare and may have a similar presentation. Their clinical diagnosis is difficult. Colour Doppler ultrasonographic findings should reveal neither flow nor communication between the popliteal vein and the lesion in either case. These findings may help differentiate between venous aneurysms and a Baker's or tibiofibular cyst.

Varicose veins are easily distinguished, being complex and elongated. In such cases, colour Doppler ultrasonography should reveal a clear communication between the lesion and the superficial vein system or through an incompetent perforating vein. In the case of a popliteal artery pseudoaneurysm there should be a localisation of the lesion within the popliteal artery, accompanied by arterial pulsations within the lesion (depicted by Doppler waveform), and the popliteal vein should not be involved. Finally, in the case of a popliteal traumatic arteriovenous fistula there is a communication between the popliteal artery and popliteal vein through the lesion, depicted using colour Doppler ultrasonography. Popliteal traumatic arteriovenous fistula is characterised by continuous turbulent flow.

## Conclusion

Popliteal venous aneurysms are rare conditions but are potentially more common than usually thought in daily practice. This case report is interesting because the diagnosis was made before the occurrence of any thromboembolic or other major complication. The physician's awareness, atypical local symptoms deserving prompt clinical explanations and access to ultrasound examination enabled early diagnosis of this case.

## Competing interests

The authors declare that they have no competing interests.

## Consent

Written informed consent was obtained from the patient for publication of this case report and any accompanying images. A copy of the written consent is available for review by the Editor-in-Chief of this journal.

## Authors' contributions

EKS, SK and CL conceived of the idea, designed and prepared the first outline of the manuscript, and revised its final version. IP carried out the ultrasound examination and provided technical content information. DK, NA and ET collected the available literature information and performed the review of the patient's medical record with helpful comments on the discussion. EKS prepared the point-by-point reply with contributions from CL. All authors read and approved the final manuscript.
